# Understanding Indigenous Australians’ experiences of cancer care: stakeholders’ views on what to measure and how to measure it

**DOI:** 10.1186/s12913-018-3780-8

**Published:** 2018-12-19

**Authors:** Monica Green, Kate Anderson, Kalinda Griffiths, Gail Garvey, Joan Cunningham

**Affiliations:** 1 Wellbeing and Preventable Chronic Diseases Division, Menzies School of Health Research, Charles Darwin University, Casuarina, NT 0811 Australia; 2 Faculty of Health Sciences, University of Sydney, Camperdown, NSW 2006 Australia; 3 Centre for Big Data Research in Health, University of New South Wales, Sydney, Australia; 4 Menzies School of Health Research, Level 1, 147 Wharf Street, Spring Hill, QLD 4000 Australia

**Keywords:** Indigenous, Aboriginal, Australia, Cancer, Patient experience, Measurement, Person-centred care

## Abstract

**Background:**

Disparities in cancer outcomes amongst Indigenous Australians reflect a pattern of reduced access to and engagement with health services. A growing emphasis on patient-centred care has increased efforts to measure patient experiences, but it is unclear whether existing approaches: a) assess the most critical aspects of care that shape the experiences of Indigenous people with cancer; and b) facilitate the engagement and participation of Indigenous people with the measurement of care experiences.

**Methods:**

Two rounds of semi-structured interviews and focus groups were used to elicit stakeholders’ views on priorities for measuring the cancer care experiences of Indigenous cancer patients and on the acceptability of various methods for capturing such information. Participants included Indigenous people affected by cancer (*n* = 17), health professionals (*n* = 28) and individuals in both groups (*n* = 7). Recruitment occurred through a national web-based network and through four cancer services in urban and regional areas in three jurisdictions across Australia.

**Results:**

Several aspects of cancer care were identified as critical in shaping Indigenous patients’ experiences. Key themes included: feeling safe in the system; importance of Indigenous staff; barriers to care; the role of family and friends; effective communication and education; and coordination of care and transition between services. Those participants affected by carers’ wellbeing and palliative care strongly advocated for the importance of these topics. Participants expressed support for a face-to-face interview with a trusted person as the most appropriate means of collecting cancer care experience information.

**Conclusions:**

While existing experience measurement tools would partially capture some important aspects of care, other critical areas would likely be missed. Appropriate tools and approaches, developed by and with Indigenous people, are urgently needed to determine the extent to which health services are meeting the needs of Indigenous people with cancer, and to identify areas for action to improve these services.

## Background

The disparity in the burden of cancer between Aboriginal and Torres Strait Islander people (hereafter respectfully referred to as Indigenous Australians) and non-Indigenous Australians is well-documented. Although there is variability across different cancer types, overall cancer incidence and mortality are higher for Indigenous Australians than for non-Indigenous Australians [1]. Cancer is the second leading cause of death among Indigenous Australians, and cancer survival is significantly lower than for non-Indigenous Australians [1]. Indigenous Australians have lower cancer screening rates [1], present at a later stage at diagnosis [1–3] and experience reduced treatment uptake in comparison to non-Indigenous Australians [3]. Poor cancer outcomes occur within a wider socio-political and historical context of racism and discrimination, social exclusion, dispossession, and forced removal of children from their families [4, 5].

Previous studies have identified a range of barriers to Indigenous Australians engaging with treatment and receiving optimal cancer care, including: alienation in the hospital environment [6, 7]; communication difficulties with health professionals [7, 8]; a lack of patient navigators [4, 9]; reduced health literacy [4, 7, 10]; lack of access to support from an Indigenous care provider [4, 11–13]; logistical impediments [14]; and inadequate linkages with primary care [4, 14–16]. Studies have also identified that respect for individual patients and their cultural perspectives is a key driver of Indigenous patients’ engagement with treatment [13, 14, 16, 17].

The Picker Institute developed eight principles of patient-centred care; respect for patients’ values, preferences and expressed needs; coordination and integration of care; information and education; physical comfort; emotional support and alleviation of fear and anxiety; involvement of family and friends; continuity and transition; access to care [18]. Patient-centred care is one of three core principles of the Australian Safety and Quality Framework for Health Care, which was endorsed (using the term ‘consumer centred care’) by Australian Health Ministers in 2010 [19]. Incorporation of patient-centred principles into cancer care services requires an understanding of how cancer patients experience their care [19–21]. This study will examine patient experiences as guided by the Picker Principles [18], rather than patient satisfaction or needs, as satisfaction is reliant on patient expectations and does not necessarily take into account good quality care. In the case of Indigenous Australians, health is considered more broadly than just the health of the individual; it includes the social, emotional, cultural and physical wellbeing of the individual’s whole community [22], and this has important implications for how Indigenous people experience care. This is reflected in Australia’s *National Aboriginal and Torres Strait Islander Cancer Framework*, which states that quality cancer care should be ‘*person centred so that the whole person (including family and cultural role) is considered, and the psychosocial, cultural and supportive care needs and preferences of Indigenous people are addressed across the continuum of care*’ ([23] .p6). The *Framework* also recommends the collection and analysis of data about Indigenous patients’ experiences of care, to enhance the capacity of cancer services to deliver integrated care that meets the needs of Indigenous people.

While this might sound straightforward, challenges in measuring patients’ experiences in general, and translating them into health system improvements are well-documented [19, 24–29]. Assessment can be based on a ‘pathway approach’ or a ‘service-centred approach’; while the former is intended to reflect a patient’s entire experience across multiple services, it is much more challenging to capture than an approach based on a single service [30]. Similarly, while surveys are easier to administer and process than narrative approaches, they can miss important details and contextual information that enable improvement-oriented action [31], and may exclude some population groups, such as those with low literacy [32] or limited English [19]. In addition, some research suggests that surveys may miss the key drivers of patient experience [33]. Thus a ‘one size fits all’ approach is unlikely to be successful [19, 32, 34], and a variety of approaches may be necessary to obtain a full picture of patients’ experiences of care [25]. A critical issue regardless of approach is the acceptance of patient feedback by health staff [35]; Roland [28] noted the importance of determining not only what should be measured and how it should be measured, but also *what difference* the measurement will make.

Interest and activity in health care experience measurement has increased in Australia in recent years [19, 27, 36, 37], and a variety of patient experience tools have been developed. While many tools have been based on principles of patient-centred care, reliability and validity varies across studies [25] and, as a result, there is little consistency and comparability across institutions and jurisdictions [19, 27]. There is also growing recognition that different approaches are needed to adequately capture and understand the perspectives and experiences of Indigenous patients [38–40], but there is little empirical evidence to date about how best to proceed. This complexity in patient experience measurement in general, together with evidence of poor cancer outcomes for Indigenous people and the fragmented nature of cancer care in particular, makes assessing Indigenous cancer patients’ experiences more challenging and urgent.

The purpose of this study was to a) identify the key components of patient experience that should be included in any experience of care measurements for Indigenous patients with cancer; and b) elicit participants’ views on the appropriateness and likely acceptability of various data collection approaches for this patient group, from the perspectives of Indigenous people affected by cancer, and health professionals involved in care provision to Indigenous patients with cancer. This information will provide important evidence to guide the development of tools and approaches to measure Indigenous Australian cancer patients’ experiences of care and, ultimately, to drive system improvement.

## Methods

This study received ethics approval from the Human Research Ethics Committees of the overseeing institution (Northern Territory Department of Health and Menzies School of Health Research (Menzies), Project Number 2015–2523), the Aboriginal Health and Medical Research Council of NSW (Project Number 1160/16) and each participating site. Guiding principles specific to research with Indigenous Australian communities informed the implementation of the study [41–44]. The study was endorsed by the Menzies Indigenous Reference Group, and two experienced Indigenous researchers were part of the five-member study team.

### Recruitment and participants

Participants were recruited between May and November 2016 from the National Indigenous Cancer Network (NICaN – a web-based network of interested individuals and organisations) and from cancer care services (three public hospitals and one regional health service) located in three Australian jurisdictions (Victoria, New South Wales and the Northern Territory). Purposive sampling was used to select study sites to reflect a variety of settings that treated enough Indigenous people with cancer to meet recruitment targets.

NICaN participants were recruited through the website or by personal referral, followed by direct contact with a study team member who explained the study and obtained consent. Potentially eligible participants from cancer care services were initially informed about the study by site staff; if permission was granted, these individuals were then contacted by a member of the study team and given detailed study information. Written informed consent was obtained from all participants. Interviews were conducted by one of two authors (MG, KG) or, in one site, by a trained local interviewer who was known to the interviewees.

Adults aged 18 years and over were eligible if they were: a) **Indigenous people affected by cancer**, including Indigenous people diagnosed with cancer and those who have cared for someone diagnosed with cancer; and/or b) **health professionals** (both Indigenous and non-Indigenous) whose work related to the care of Indigenous people diagnosed with cancer, including a broad range of clinical, supportive care, quality improvement and supervisory roles. Individuals deemed too unwell to participate were excluded; this determination was made by the staff in each of the recruiting centres, with no involvement from study personnel.

### Data collection

**Fig. 1:**
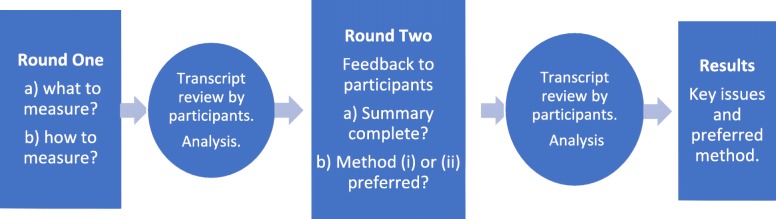
Study overview

As shown in Fig. 1, two rounds of semi-structured, in-depth interviews or focus groups were offered to participants, either face-to-face or by telephone, depending on geographical restrictions. All sessions were audiotaped with the participant’s consent and transcribed; participants received a copy of their transcript and were invited to clarify or correct the transcript and provide new comments if they wished.

Round One interviews were introduced with the definition of ‘good quality cancer care’ from the *National Aboriginal and Torres Strait Islander Cancer Framework* ([23] .p6), to highlight the ‘person-centred’ orientation of the study team. A person-centred approach was taken for several reasons, including: 1) the comprehensive adoption of this approach to health care by the Australian government [19]; 2) reiteration of person-centred care in the *Framework* [23]; and 3) the alignment of person-centred care with the Indigenous conceptualisation of health, as described above*.*

Round One interviews were structured around a short set of questions and prompts, which were formulated through a review of the literature, knowledge of current activity in the area, and pilot interviews with Indigenous and non-Indigenous people. In addition to basic demographic information, the interviews aimed to elicit: (a) *which components* of patient experience should be measured; and (b) *how* these components should be measured in the Indigenous community. To prompt discussion about various survey methods and styles of response, the UK National Health Service’s ‘National Cancer Patient Experience Survey’ [45], was presented to participants. This survey has been the basis for population-based experience of care surveys undertaken in Australia [38, 46, 47]. As our aim was to inform the development of systematic methods of measurement, population-based surveys were our starting point, and we wished to understand stakeholder views on this approach.

Round Two sessions were open to Round One and new participants, and aimed to: a) identify and correct gaps or omissions in the Round One summary; and b) in relation to Round One results, review and discuss the design, content and response categories of existing surveys and a set of questions developed by the study team, then suggest how best to measure experiences of care in this population group between the following options: (i) a tick box survey approach; and (ii) an approach based on the Here and Now Aboriginal Assessment (HANAA), a social and emotional wellbeing screening tool which covers a range of domains discussed with a client, then rated as ‘Problem’ (with a summary of the issues recorded by the interviewer) or ‘No Problem’ [48].

### Analysis

Transcripts from all participant sessions were de-identified and imported into NVivo11 software (QSR International Pty Ltd., Doncaster, Victoria) to manage the data. An iterative approach was taken to the analysis, whereby a Grounded Theory methodology was used to generate a coding system and develop themes. Detailed and repeated coding of transcripts by two researchers (MG, KA) was undertaken, and considerable effort was made to maximise inter-rater reliability and inter-coder agreement, guided by previous qualitative research [49].

Round Two data were repeatedly reviewed to determine: if the themes from Round One were sufficiently comprehensive; and participants’ preferred measurement options. Summary data has incorporated this feedback.

## Results

### Participant characteristics

A total of 52 participants were included in the study (Table 1). Forty-eight people were interviewed in Round One, including 26 Indigenous people (54%). Seventeen participants were Indigenous people affected by cancer (‘CaAff’), 26 were health professionals (‘HP’) (including 4 Indigenous HP who did not report being affected by cancer outside their work), and 5 people were both Indigenous people affected by cancer and health professionals (‘Both’). Participants ranged from 22 to 73 years of age, and the majority were female (81%). Thirty-two Round One participants (67% overall; 74% for HP and 59% for CaAff) completed an interview or focus group in Round Two. Four new participants (all Indigenous) joined the study in Round Two, including two health professionals and two people who were both affected by cancer and health professionals.

**Table 1:** Participant demographic characteristics

	CaAff(a)	HP(b)	Both(c)	Total
	*n* = 17	*n* = 28	*n* = 7	*n* = 52
Age (years)				
20–39	2	3	1	6
40–59	10	16	5	31
≥ 60	5	9	1	15
Gender				
Female	11	25	6	42
Male	6	3	1	10
Recruitment source				
Urban sites	2	15	1	18
Regional site	7	10	3	20
NICaN(d)	8	3	3	14

(a) CaAff: An Indigenous person affected by cancer, either diagnosed or as a carer

(b) HP: A health professional whose work relates to care of Indigenous people with cancer

(c) Both: An Indigenous person affected by cancer who is also a health professional whose work relates to care of Indigenous people with cancer

(d) NICaN: the National Indigenous Cancer Network

Fifteen of 24 participants (62%) affected by cancer had been diagnosed with cancer, the majority within the last 5 years (*n* = 8), and almost all within the last 8 years (*n* = 14). Breast cancer (*n* = 5) was the most common cancer type, followed by prostate and bowel cancer (*n* = 2 of each type). Two people had experienced multiple cancer diagnoses. Of the 24 Indigenous people affected by cancer (including ‘CaAff’ and ‘Both’), 5 resided in major cities, 8 lived in inner regional areas, 9 in outer regional areas and 2 in remote areas. Of the 35 health professionals (including ‘HP’ and ‘Both’), ten were Indigenous care providers (29%) (e.g. Aboriginal Liaison Officers (ALO), Aboriginal Health Workers (AHW)), eight were social workers (23%), and six were nurses (19%), with a range of other professions represented.

Average interview length for Round One was 28 min. Although offered to all participants where feasible, only two group sessions were held (in Round Two). Overall, 84 sessions were held: over half (*n* = 49; 58%) were phone interviews; 27% (*n* = 23) were face-to-face and 14% (*n* = 12) were discussion groups. Two cancer-affected participants made minor changes to their interview transcripts. Due to the small numbers of participants in each group, age and gender of participants are not reported with individuals’ quotes, to protect confidentiality.

### Overall context as described by participants

The context in which cancer occurs is an important factor in shaping how cancer care is experienced. Key contextual factors identified by participants included past and present experiences of racism and discrimination, the underlying patterns of illness in the Indigenous population, health system characteristics and the varied life circumstances of patients. Many participants referred to the lack of open discussion about cancer (‘*the ‘C’ word’;* 512 CaAff:) in the Indigenous community, for reasons including stigma, large amounts of existing stress, and different ways of dealing with challenges. Although many participants indicated that awareness of cancer is gradually increasing, they stressed the need for continued education programs to counter myths about cancer and its treatment. Many participants believed that *‘There needs to be more conversations, more yarns about the cancer journey and what to do and resources’ (304 CaAff)* to help people through their experience. (‘Yarning’ is a widely used term for an Indigenous style of conversation and storytelling [50]). A pervasive message across study participants was that Indigenous people are not all the same and need to be treated as individuals; it was recognised that a person’s life circumstances and background can impact significantly on their engagement with treatment and their need for support, and these should be considered by health services and staff, in addition to broader cultural issues.

### Key issues impacting Indigenous patients’ experience of cancer care

Several themes emerged as central in shaping how care is experienced by Indigenous Australians with cancer: the themes regarding ‘what to measure’ are presented in Table 2 and are discussed below. Our analysis of participants’ responses regarding ‘how to measure’ patient’s experiences for this patient group is also outlined below.

**Table 2:** Themes and priorities raised by cancer-affected participants and health professionals, with illustrative quotes

Theme	Common priorities amongst participant groups	Participant quotes
Feeling safe in the system	Compromised trust in the system and individual staff; impact of colonisation and institutional racism; hospital surroundings in the context of cultural safety; positive impact of a trusting relationship with staff.	• '*'We had a connection because I built up a relationship with her that I could actually talk to her openly and honestly about*.’ (302 CaAff). •‘*The issue of decision-making is one where everyone says it’s your own decision but the reality is that it’s not actually. Because of your economic situation, your cultural timidity or you’re culturally intimidated by this dominant white medical culture*.’ (511 CaAff).
Importance of Indigenous care providers	Access to an ALO/AHW important for whole pathway, particularly if patient away from home or lacking family support; lack of Indigenous health staff, which can positively influence a patient to stay in treatment; Support for Aboriginal Community Controlled Health Organisations (ACCHOs).	• ‘*this big health service was here. But where was my people*?’ (305 CaAff). • ‘*part of you’; ‘more approachable in the community*’ (511 CaAff, referring to ACCHOs).
Barriers to care	Logistical barriers such as finance, accommodation and transport, more acute for those people receiving treatment away from home; acknowledgment that programs exist to address shortfalls, but they are not applied comprehensively.	• '*'So if a patient needs six weeks of daily chemo and needs to stay there, that’s a big financial burden, especially if they’ve living in the regions and they need to come into the city for appointments, petrol money – you know, that was a big issue too'*. (504 Both). • *'Lot of Aboriginal people won’t go because they’ve got no money for travel; they’ve got no money for food*'. (503 CaAff).
The role of family and friends	Inclusion of family and friends was seen as crucial by all participant groups and contributed to relieving fear and anxiety; accommodating family (both literally and figuratively) in the hospital setting.	• ‘*You know they may be the decision-maker in the family and things like that. So while they’re away having their treatment, the family…breaks down…And then they’re coming in, …to the patient and they’re bringing those worries from home in with them...*’ (504 Both).
Effective communication and education	Reduced ability to absorb information following a cancer diagnosis; appropriate language; relationship building; listening to patients; determining who to communicate with in family; unconscious bias; need to feel safe to ask questions.	• ‘*repeating that information’s important because all the information–like you only really get 10 % of it – you’re just shocked and end of the world and stressed*’ (507 CaAff).
Coordination of care/navigation of the system and transition between services	Coordination of care was commonly reported as lacking and being of crucial importance. The need for assistance to navigate through complex treatment regimens and systems, was commonly (though not exclusively) reported in relation to the transition into primary health care following treatment.	*• ‘I found that I was let down with one of the main services that I really…depended on…(after) being discharged from hospital. I had a bad experience. I was left to fend for myself. I had to maintain my house ….which resulted (in) me getting infected and.. a lot of follow-up with doctors and medication, which could have been avoided..’ (305 CaAff).*

Legend: *CaAff* Indigenous person, cancer affected; *HP* Health professionals; *Both* Indigenous person, cancer affected and a health professional

#### Feeling safe in the system

Cancer-affected participants commonly spoke about their interactions with the health system and clearly expressed a need to feel safe within the system in order to engage with treatment. These participants spoke of feeling intimidated, of ‘*cold’* interactions, of the ‘*medical monster*’, ‘*dominant white medical culture*’ (511 CaAff) and of personal stories impacting on whether they trusted the system enough to take part in treatment. As one participant put it: *‘When I walk into a hospital today, I’m very wary of who …can I trust. Who can I talk to?* (512 CaAff) The impact of institutional racism and history on the willingness and ability of Indigenous people to engage with the health system was evident throughout the cancer-affected participants’ interviews: ‘*it’s very hard for Aboriginal people to trust… after what they’ve been through over the two hundred years’* (503 CaAff).

Health professionals also recognised the importance of history, in particular the impact of the Stolen Generations (a term referring to “the generations of Indigenous children that were forcibly removed from their families by compulsion, duress or undue influence, as a result of protectionist and child welfare laws, practices and policies in place in Australia for most of the 1900s”. [51]). *‘And also they’re still very distrustful from the system. I don’t think it’s our fault. It was our previous system you know, like the Stolen Generation. It’s still very much a big consideration even nowadays.’* (315 HP). Health professionals talked about the importance of developing trusting relationships to overcome this: *‘I think probably the biggest thing is trust and in palliative care it frequently takes several visits to develop that trust. It’s still really important with every individual that we really actively try and engage with the patient and their community.*’ (407 HP) However, this trust could be fragile; if broken it could be critical in defining the person’s experience of care: *‘And they said, “Oh, Aboriginal people don’t burn when they have radiation”. And that’s an outright lie …Because I was burnt red raw from radiation.'* (503 CaAff).

A further issue, raised primarily by health professionals, was whether a person identifies as Indigenous within the health care system; this was seen as a reflection of the extent to which a person felt culturally safe in interactions with the health system.

#### Importance of Indigenous care providers

All participant groups recognised that the presence or absence of an Indigenous care provider is a crucial aspect in shaping the experiences of cancer-affected participants, who described themselves as being ‘*not as guarded*’ (302 CaAff) and feeling freer to ask questions without feeling silly, with an Indigenous person*.* As one participant recalled, *‘It was important to talk with an Aboriginal person – far more important than …the social worker for me.’* (303 CaAff). Access to an Indigenous care provider was not universal, however, and the paucity of Indigenous health care providers in the system was described as ‘*where the big breakdown is …where you fall through the loop.’* (512 CaAff).

Health professionals also spoke about the benefits to staff of having an Aboriginal Liaison Officer (ALO) and/or Aboriginal Health Worker (AHW) in the service, including: helping staff understand why a patient/family member may be responding in a certain way; enabling the patient to trust enough to explain their concerns; and facilitating better linkages with services outside the hospital. *‘[O]nce they’ve seen that I’ve been able to work with the liaison officer, I’ve been able to build really strong relationships after that…..So it’s helped me to be introduced as a safe person...’* (HP 205).

#### Barriers to care, particularly if receiving treatment away from one’s Country

Among cancer-affected participants, key challenges, especially for those needing to travel for treatment, included: logistical difficulties and costs associated with transport, accommodation and food; separation from family and support networks during a very stressful time; and costs associated with bringing family support to patients.

*‘Going away and being treated – that’s the biggest thing because you know one of the most important things when you’re not well is to have your home. I think it’s better to be treated at home because you might not have the people around you as you’re going through treatment’* (304 CaAff).

Being away from one’s own Country or traditional lands, including the possibility of dying off Country, was also a particular source of distress for some participants.

*(Aboriginal care provider) really understood where I was coming from being off Country. They understood my fears about being off Country and especially dying off Country – what would happen to my spirit, how would they treat my body and the aunties were able to explain the process of what happened if I did pass off Country and what would happen to my body*.’ (303 CaAff).

These issues, on top of the emotional strain of cancer diagnosis and treatment, were clear sources of anxiety for many, especially for those living outside of major metropolitan areas. *‘…older people, sickly people – how do they expect them to get on the train and then find their way home? So there has to be better systems in place.’* (301 CaAff).

The sense of frustration at not knowing where or how to access information or support for these basic needs was also common. These stresses were compounded by the often rapid diagnosis and urgent start of treatment for many Indigenous patients, leaving little time for planning and coming to terms with the transition to urban hospital settings.

Health professionals clearly indicated their view that patients are more likely to engage with continuing treatment if they are provided with practical support such as accommodation for family, transport, and help to access financial support.

#### The role of family and friends

The role of family and friends in providing additional support was commonly mentioned, with a broad range of support types highlighted, such as emotional, spiritual, practical, advocacy, home care, information provision, moral support and gatekeeping. *‘I think it would be a terrible journey to not have any loved ones around you as you’re going through that time.’* (304 CaAff).

A diagnosis of cancer resulted in intense emotions including grief and shock, not only for patients, but also for family and friends. While family and friends were seen as critical in helping to manage fear and anxiety and providing practical and logistical assistance, it was recognised that they, too, needed support. *‘…when you support an Aboriginal person, you’re not just supporting that person. You need to support the family as well along their journey.’* (408 Both).

Challenges for cancer-affected participants included: needing to keep the family informed with what can be distressing and sensitive information; dealing with family conflict; and other stressors in community life impacting on how the cancer pathway is experienced. The role of the patient within the extended family and the resultant extended impact of the diagnosis were spoken about by many cancer-affected participants. The patient may be the main person in the extended family with a car/licence, for example, or be the key person providing other support to the extended family, which further extends the impact of the diagnosis and influences the type and level of support required to sustain engagement with treatment.

#### Effective communication and education

The need for effective communication and education was raised by both cancer-affected and health professional participants. Key points relating to information provision included: the importance of using accessible and appropriate language; using diagrams or drawings to aid comprehension; limiting the amount of information provided at any one time; considering the optimal timing of information provision; and recognising the need to repeat information over the course of the cancer journey.

Some participants stressed the need to provide consistent information to both patients and their family members, as confusion and distress can occur if all affected parties are not kept informed. At the same time, it was critical to ensure that patients and families were *‘comfortable hearing’* (HP 407) what needed to be said.

The centrality of communication to the patient experience and a need for improvement in communication were commonly reported by health professionals.

*I think there are probably some specific things about communication that we need to be taught by Aboriginal patients and their families that are culturally specific and that we might not even be aware of … and we think we’ve offered an opportunity for questions or discussion but we’ve done it in a way which is actually not going to allow that person to engage with us.’* (403 HP).

The importance of relationships as a facilitator of good communication as well as quality care was also highlighted.

*‘That’s what’s making us provide as optimal care as we can is if we stop thinking we’re just treating cancer. We’re treating our patient, and what could we do that helps this particular patient through?.... that just comes with relationships and communication.’* (101 HP).

#### Coordination of care and transition between services

The need for assistance to navigate through complex treatment regimens and systems, and the challenges stemming from a lack of overall coordination of care were commonly reported by participants. *‘There’s too many people involved and they need continuity.’* (503 CaAff) The potential benefits of a designated care coordinator or patient navigator to assist patients through an often highly-fragmented cancer care system were recognised: *‘…certainly with the Aboriginal women that we see who come in from regional areas, I think that navigator is an extremely important person in the scheme of things to coordinate care.’* (404 HP).

The absence of culturally appropriate support services during the transition between services was a significant stressor for many participants. This was commonly (though not exclusively) reported in relation to the transition back to primary health care following treatment. One participant questioned where this support was, because Indigenous people ‘*will never ask*’ (502 CaAff). Health professionals were not necessarily well-placed to answer this question, however: *‘…we don’t always have a good sense of what’s on the ground (outside hospital) and clients aren’t able always to tell us either.’* (203 HP).

#### Other issues

Although a negative experience with pain management was mentioned by two cancer-affected participants (one of which had catastrophic consequences), in general, pain management and physical comfort did not feature strongly in the reported experiences of any participant group.

Participants referred to the hospital environment and surroundings only in the context of cultural safety, including: the intimidating nature of the hospital environment; the presence or absence of Indigenous artwork and flags; the ability to engage in cultural practices, such as smoking ceremonies; space for multiple visitors in hospital (without judgment); and access to garden areas, enabling people to feel more relaxed, able to talk and to receive information.

While most participants found the summary of key themes identified in Round One to be comprehensive, a few participants indicated that greater attention was needed for two issues: 1) caring for carers; and 2) palliative care. It was noted that carers undergo sustained periods of dealing with multiple stressors, combined with a lack of attention to their needs and their welfare and little-to-no follow-up, and that this contributed to a sense of being disregarded once the person being cared for had passed away or had reached a less acute stage. *‘…the carer is the one that carries the load. You know they’re the ones that are looking after the sick person as well as trying to manage family.’* (103 Both).

While palliative care was not discussed by most participants, those who mentioned it expressed strong views about the importance of appropriate end-of-life care.

*‘The palliative care journey, the end of life, and respecting patients’ choices, that’s really new for our mob…, because it’s such a taboo when there is a passing of our elders or a community member, that it’s more out of respect that we don’t mention (it), so, it’s just breaking down the barriers, about how we can actually have more of that conversation with our mob …and how important it is that we respect at the end of your journey what would you like to have happen.’* (318 HP, Indigenous).

### Participants’ views on ‘how to measure’ experiences of care

In Round One, most participants indicated that a face-to-face interview with a trusted person would be the best approach to measuring Indigenous patients’ experiences of care. There was a clear preference among all groups for an opportunity for ‘yarning*’*, with several people suggesting a group or workshop setting. The issue of trust, in relation to who is doing the survey and why, was also raised frequently. A survey-style approach was commonly supported with caveats, such as the option to be supported or guided to complete the survey, or to have a phone contact for support. Most participants suggested that a preference for paper versus touchscreen surveys would be guided by a person’s age, with older people possibly preferring paper, or having a guide if using a touchscreen. Some participants reported previous experience of a touchscreen not working at all, even with guidance. There was very little support for emailed, online or mailed surveys, with many indicating that electronic surveys are easily ignored.

The most common view regarding the length of any survey was to keep it short. The timing was suggested to be once the patient is ‘settled’ or at different phases of the pathway. In terms of the style of responses presented in a survey, many participants advocated inclusion of open-ended questions. The importance of including patients from regional and remote areas was noted by many participants. The appearance of and language used in the survey needed to be seen as relevant to Indigenous people.

Regarding Round Two ‘how to measure’ feedback, although many questions in the survey approach (i) were viewed positively by participants, method (ii) [‘problem/no problem’ approach], was seen as ‘open’ and ‘refreshing’ compared with the traditional survey approach, and was overwhelmingly preferred. It was also seen as more likely to result in greater participation.

## Discussion

This paper presents the views of Indigenous Australians affected by cancer and of health professionals, regarding how care is experienced by Indigenous people with cancer. Although many issues raised by participants in our study are common across populations, the focus of this report is on the issues that emerged from the analysis as being: a) specific to the Indigenous population; b) prioritised differently from the general population; or c) experienced in a unique way by Indigenous Australians with cancer. This information provides the basis for developing a method to describe and measure those experiences in a systematic way. The study found that Indigenous Australians report significant difficulties in accessing and engaging with cancer care, many of which may be missed by commonly used approaches to measuring patients’ experiences. While many of the issues reported align with one or more of the principles of person-centred care, the strong emphasis by our participants on issues relating to respect, trust, and cultural safety, and the presence of important contextual issues, indicates that alternative approaches are warranted. While a comprehensive analysis of existing measurement tools is outside the scope of this report, the failure to measure and amend the deficiencies of care reported here are likely to have greater significance for Indigenous people, given their already reduced engagement with and access to the health system and the wider context of racism and discrimination.

The findings of the current study are generally consistent with prior qualitative studies in this area. Previously reported issues relating to cross-cultural care were repeatedly raised by participants in this study, including: difficulties establishing trust given the historical context [4, 9, 12]; a lack of Indigenous staff [7, 11, 12]; communication difficulties with health staff [8, 11]; the intimidating nature of the health system [4, 12]; challenges in transitioning to primary care [14]; and the lack of understanding of cultural issues, such as being away from Country and the importance of family, as well as the importance of respect for the individual and his or her cultural perspective [8, 11, 12, 16]. A New South Wales Bureau of Health Information survey [38] of people who had been hospital inpatients found that Aboriginal respondents were less positive than non-Indigenous respondents about experiencing respect relating to culture, dignity and privacy, which is consistent with the current study. Our results provide more detail around cultural issues, including cultural safety, which contribute to reduced engagement with and access to the health system for Indigenous people.

Access to an Indigenous care provider was commonly reported as a crucial, yet often lacking, aspect of care, echoing findings in previous studies [7, 9, 11, 13, 14]. Indigenous care providers promote culturally safe service delivery and help to bridge the cultural gap [17]. Our findings suggest that, while Indigenous people can and do form trusting relationships with non-Indigenous health professionals, access to Indigenous care providers substantially improves Indigenous people’s experiences of cancer care.

Logistical barriers to care for Indigenous people, particularly for those receiving treatment away from home, have been well documented [8, 11, 12, 15, 17] and are reiterated in the current study. Such barriers are modifiable and, though some participants indicated that efforts are in place to address many of these barriers, ongoing monitoring is required to ensure the potential benefits are realised. The issue of being away from one’s Country to receive treatment is significant for Indigenous people, not only because of logistical barriers, but also due to the loss of emotional and cultural support networks and the loss of spiritual and other benefits of connection to Country during a stressful and traumatic time.

Participants in the current study spoke about the intense emotional strain following cancer diagnosis and treatment, commonly exacerbated by pre-existing stressors and a historically-based mistrust of the health system. This strain was centrally important in shaping patients’ experiences of care, overshadowing many other aspects measured in existing patient experience measurement tools, such as the physical environment. Participants almost universally expressed that this stress was ameliorated by a range of different types of support from family and friends.

Communication, education, and information provision are key domains in experience of care measurement [19, 24, 27, 36]. Among our cancer-affected participants, these domains were commonly connected to respect, trust and an interpersonal relationship. The interaction of these issues appeared pivotal to effective patient education. Trust has also been identified as part of supporting quality improvement [52] and addressing cultural acceptability [17], and is improved with patient navigation [9], which has implications for experience measurement.

Reinforcing previous studies [7, 9], participants in our study commonly felt that coordination of care, particularly around the transition into primary health care, was unsatisfactory, and this requires more detailed exploration in future measurement activities for this patient group. The lack of awareness of post-discharge support services and the perceived lack of cultural safety of these services were commonly held concerns. Problems with the transition from hospital to home care have also consistently been highlighted in previous qualitative studies [4, 7, 12]. According to a recent Bureau of Health Information survey in NSW, 83% of Aboriginal hospital patients reported needing their family and home situation taken into account upon discharge and 72% reported needing services after discharge, compared with 77 and 61% respectively among non-Aboriginal respondents [38]. Although the survey was not cancer-specific, it highlights the relatively high level of post-discharge support required. In the context of poorer health outcomes, greater comorbidity, and reduced engagement with health services in this population, a failure to respond to such needs may have a critical impact on Indigenous patients’ experiences of care.

These results have a number of implications for the measurement of Indigenous patients’ experiences of care. To improve patient experience and engagement, the ability to measure and enhance Indigenous patients’ sense of feeling safe in the system is paramount, including the impact of being away from one’s Country for treatment. Access to an Indigenous care provider is readily measurable and modifiable and this should be a routine part of measuring the experiences of care of Indigenous patients with cancer. Closer attention to the role of family and friends is warranted when measuring experiences of care of Indigenous people with cancer, including monitoring the wellbeing of carers, particularly in palliative care situations. Our results suggest that the issues of communication, education and information provision must be considered in the context of trust and interpersonal relationships when measuring the care experiences of Indigenous cancer patients. The issues of caring for carers and palliative care were not reported by all participants, however these are important aspects of care and warrant assessment in their own right.

Several components of care included in existing experience of care measurement instruments [45] did not feature prominently in our participant interviews, such as dietary issues, safety issues and the physical environment. The latter two were only mentioned by our participants in relation to cultural safety, such as space for relatives to visit, or having a place for smoking ceremonies, reinforcing previous research [13]. Pain control was mentioned by only two participants, though one of these endured catastrophic consequences. Despite this lack of prominence, patient safety remains a critically important aspect of quality care and therefore should be monitored alongside other aspects of care. The importance of this issue is highlighted by evidence about differences in pain management across Indigenous and non-Indigenous populations [53].

Challenges surrounding data collection methodologies for measuring the experience of care of Indigenous people have been identified [32, 36, 38, 40, 54]. While some research has recently been reported [9, 13], increased attention to the area is required [27] to achieve a systematic approach. Participants in the current study showed a clear preference for measurement methods that enable telling their story to a trusted person or completing a survey with access to support from an appropriate person, rather than a mailed or electronic survey without support. One recent patient experience survey (not cancer-specific) attained a 21% response rate using mailed surveys with Indigenous people [38]. Aside from limitations associated with low response rates, it is questionable whether components of care that are crucial to Indigenous people could be adequately captured using such an approach. In any case, these methodological issues are in addition to the key challenge facing experience measurement in any population: translating data into action [24, 32].

Despite recent and current work on experience measurement, in general there remains a lack of systematically collected data on the issues facing Indigenous cancer patients identified in the current study. Large-scale feasibility remains a challenge, particularly given the compromises of a survey style approach, which may miss the key drivers of patients’ experiences [33] by not adequately capturing narrative [31, 54]. We recognise that the assessment of whether person-centred care has been delivered requires multiple avenues of exploration [19, 25, 32, 34]. This study provides details on the priority areas and measurement methods which may be important and acceptable to Indigenous people. Many of the problems identified in this study are amenable to being measured and addressed if appropriate questions about patient experiences are asked and acted upon. Although additional resources may be required to measure Indigenous patient’s experiences in ways that allow them to tell their story to a trusted person, this study suggests that the investment would be worthwhile due to a greater likelihood of eliciting the care experiences that would enable effective change. Development of suitable methodologies must entail leadership by and partnership with Indigenous stakeholders to ensure acceptability to and appropriateness for Indigenous people [39], as well as the involvement of care providers and service managers, to ensure that the data collected are both actionable and acted upon to improve services.

It is clear that when attending health services, some Indigenous people are not asked whether they identify, or are choosing not to identify themselves, as Aboriginal or Torres Strait Islander [55]. Health professionals expressed concerns that not all cancer-affected Indigenous people were receiving appropriate support because of this, echoing concerns reported in other studies [9]. Close examination of this problem is not within the scope of the current study and its effect on the study results is unknown.

There are some limitations in the design of this study. While rural and urban populations were included in our sample, Indigenous people living in remote areas were under-represented. Females were over-represented, which is frequently the case in studies of this nature and 60% of participants were aged 40–59 years. The voluntary nature of the study means that the voices of those who were unable or unwilling to ‘speak up’ may not have been captured, and the study did not include those who had not presented, refused treatment or had disengaged from the health system. While including the views of carers and health professionals may have mitigated these limitations somewhat, it is likely that the severity and extent of the issues identified may understate the real picture. However, the consistency of responses about key aspects of experience makes it unlikely that important areas have been missed. Participants’ degree of familiarity with the interviewer may also affect responses, however the effect of this is difficult to determine.

## Conclusions

Many issues identified in this study reinforce factors consistently highlighted previously: feeling safe in the system; access to Indigenous staff; logistical impediments to accessing care, particularly if away from home; the importance of family involvement; communication and education difficulties; and problems transitioning between services and a lack of coordination of care [4, 7, 14]. While some issues are partially captured in patient-centred care principles which underpin existing experience of care instruments, the specific cultural, historical and socioeconomic contexts in which Indigenous Australians experience cancer care demands the reframing of these approaches, to more effectively capture the values, preferences, practices and needs of this patient group. Further research, led and guided by Indigenous people, is needed to develop appropriate methods to identify deficiencies in experiences of care of Indigenous people with cancer, focusing on modifiable issues and effective feedback mechanisms. This will enable health services to adapt the delivery of cancer care to better meet the needs of Indigenous people with cancer.

## Abbreviations

ACSQHC: Australian Commission on Safety and Quality in Health Care
AHMRC: Aboriginal Health and Medical Research Council
AHW: Aboriginal Health Worker
AIHW: Australian Institute of Health and Welfare
ALO: Aboriginal Liaison Officer
BHI: Bureau of Health Information
HANAA: Here and Now Aboriginal Assessment
NHMRC: National Health and Medical Research Council

## References

[1] Australian Institute of Health and Welfare & Cancer Australia. Cancer in Aboriginal and Torres Strait Islander peoples of Australia: an overview. Cancer series no.78. Cat. no. CAN 75. 2013. Canberra Australia. https://www.aihw.gov.au/getmedia/aa938fd4-21e8-4854-9207-c70306e4f2b3/13732.pdf.aspx?inline=true. Accessed 6 Dec 2018.

[2] Condon JR, Garvey G, Whop LJ, Valery PC, Thomas D, Gruen R, Cunningham J. Aboriginal and Torres Strait Islander Australians and cancer. Cancer Forum. 2013;37(1):27–30.

[3] Cunningham J, Rumbold AR, Zhang X, Condon JR. Incidence, aetiology, and outcomes of cancer in Indigenous peoples in Australia. Lancet Oncol. 2008;9(6):585–95. 10.1016/S1470-2045(08)70150-5 18510990

[4] Treloar C, Gray R, Brener L, Jackson C, Saunders V, Johnson P, Newman C (2014). “I can’t do this, it’s too much”: building social inclusion in cancer diagnosis and treatment experiences of Aboriginal people, their carers and health workers. Int J of Pub Health.

[5] Commonwealth of Australia. Closing the gap on Indigenous disadvantage: the challenge for Australia. 2009. Commonwealth of Australia, Canberra. https://www.dss.gov.au/sites/default/files/documents/05_2012/closing_the_gap.pdf.

[6] Newman C, Butow P, Knight R, McMillan K, Treloar C, Kippax S, Eades S (2008). Cancer and Aboriginal people in Australia: a review of the literature. Crit Public Health.

[7] Worrall-Carter L, Daws K, Rahman MA, MacLean S, Rowley K, Andrews S, MacIssac A, Lau PM, McEvedy S, Willis J, Arabena K (2016). Exploring Aboriginal patients’ experiences of cardiac care at a major metropolitan hospital in Melbourne. Aust Health Rev.

[8] Shahid S, Finn L, Bessarab D, Thompson SC (2009). Understanding, beliefs and perspectives of Aboriginal people in Western Australia about cancer and its impact on access to cancer services. BMC Health Serv Res.

[9] Reilly R, Micklem J, Yerrell P, Banham D, Morey K, Stajic J, Eckert M, Lawrence M, Stewart HB, Brown A. Other CanDAD investigators, the CanDAD Aboriginal community reference group. Aboriginal experiences of cancer and care coordination: lessons from the Cancer data and Aboriginal disparities (CanDAD) narratives. Health Expect. 2018:1–10. 10.1111/hex.12687. 10.1111/hex.12687 PMC6186541 29691974

[10] Treloar C, Gray R, Brener L, Jackson C, Saunders V, Johnson P, Harris M, Butow P, Newman C (2013). Health literacy in relation to cancer: addressing the silence about and absence of cancer discussion among Aboriginal people, communities and health services. Health Soc Care Community.

[11] Kelly J, Dwyer J, Mackean T, Willis E, O’Donnell K, Battersby M, Pekarsky B. Managing Two Worlds Together: Study 3—The Experiences of Patients and Their Carers. 2011. The Lowitja Institute, Melbourne. https://www.lowitja.org.au/sites/default/files/docs/M2WT_Study_3.pdf. Accessed 6 Dec 2018.

[12] Shahid S, Teng T-HK, Bessarab D, Aoun S, Baxi S, Thompson SC. Factors contributing to delayed diagnosis of cancer among Aboriginal people in Australia: a qualitative study. BMJ Open 2016;6:e010909. 10.1136/bmjopen-2015-010909. 10.1136/bmjopen-2015-010909 PMC4893856 27259526

[13] Wotherspoon C, Williams CM. Exploring the experiences of Aboriginal and Torres Strait Islander patients admitted to a metropolitan health service. Aust Health Rev. 2018. 10.1071/AH17096. 10.1071/AH17096 29495978

[14] Shahid S, Finn L, Bessarab D, Thompson SC (2011). ‘Nowhere to room ... Nobody told them’: logistical and cultural impediments to Aboriginal peoples’ participation in cancer treatment. Aust Health Rev.

[15] Micklem JM. Self-reported health-related quality-of-life issues for Aboriginal and Torres Strait Islander patients with experience of cancer in Australia: a review of literature. Int J Evid Based Healthc. 2015;13. 10.1097/XEB.0000000000000051 26126000

[16] Tranberg R, Alexander S, Hatcher D, Mackey S, Shahid S, Holden L, Kwok C. Factors influencing cancer treatment decision-making by Indigenous peoples: a systematic review. Psycho-Oncology. 2015;25:131–41. 10.1002/pon.3900. 10.1002/pon.3900 26152813

[17] Ware V-A. Improving the accessibility of health services in urban and regional settings for Indigenous people. Resource sheet no. 27. 2013. Produced for the Closing the Gap Clearinghouse. Canberra: Australian Institute of Health and Welfare & Melbourne: Australian Institute of Family Studies. https://www.aihw.gov.au/getmedia/186eb114-8fc8-45cc-acef-30f6d05a9c0c/ctgc-rs27.pdf.aspx?inline=true Accessed 6 Dec 2018.

[18] Picker Institute. Eight Dimensions of Patient-Centred Care. 1987. https://www.picker.org/aboutus/picker-principles-of-person-centred-care/. Accessed 6 Dec 2018.

[19] Australian Commission on Safety and Quality in Health Care. Review of patient experience and satisfaction surveys conducted within public and private hospitals in Australia. 2012. https://www.safetyandquality.gov.au/wp-content/uploads/2012/03/Review-of-Hospital-Patient-Experience-Surveys-conducted-by-Australian-Hospitals-30-March-2012-FINAL.pdf. Accessed 6 Dec 2018.

[20] Australian Institute of Health and Welfare (2014). The measurement of patient experience in non-GP primary health care settings. Cat.No.WP66.

[21] Australian Commission on Safety and Quality in Health Care. Patient-centred care: Improving quality and safety through partnerships with patients and consumers, ACSQHC, Sydney. 2011. https://www.safetyandquality.gov.au/wp-content/uploads/2012/03/PCC_Paper_August.pdf. Accessed 6 Dec 2018.

[22] National Aboriginal Health Strategy Working Party. A National Aboriginal Health Strategy Canberra: Department of Aboriginal Affairs, 1989.

[23] Cancer Australia. National Aboriginal and Torres Strait Islander Cancer Framework 2015. Cancer Australia, Surry Hills, NSW. https://canceraustralia.gov.au/sites/default/files/publications/national-aboriginal-and-torres-strait-Islander-cancer-framework/pdf/2015_atsi_framework_1.pdf. Accessed 6 Dec 2018.

[24] De Silva D. Measuring patient experience. 2013. The Health Foundation, London. https://www.health.org.uk/sites/health/files/MeasuringPatientExperience.pdf. Accessed 6 Dec 2018.

[25] De Silva D. Helping measure person-centred care. 2014. The Health Foundation, London. https://www.health.org.uk/sites/health/files/HelpingMeasurePersonCentredCare.pdf. Accessed 6 Dec 2018.

[26] Gleeson H, Calderon A, Swami V, Deighton J, Wolpert M, Edbrooke-Childs J (2016). Systematic review of approaches to using patient experience data for quality improvement in healthcare settings. BMJ Open.

[27] Harrison R, Walton M, Manias E, Mears S, Plumb J (2016). Patients’ experiences in Australian hospitals: a systematic review of evidence. Aust Health Rev.

[28] Roland M (2012). Measuring and improving patient experience in primary care. Primary Health Care Research & Development.

[29] Sandager M, Freil M, Knudsen JL (2016). Please tick the appropriate box: Perspectives on patient reported experience. Patient Exp J.

[30] Gibbons EJ, Graham C, King J, Flott K, Jenkinson C, Fitzpatrick R (2016). Developing approaches to the collection and use of evidence of patient experience below the level of national surveys. Patient Exp J.

[31] Tsianakas V, Maben J, Wiseman T, Robert G, Richardson A, Madden P, Griffin M, Davies EA (2012). Using patients' experiences to identify priorities for quality improvement in breast cancer care: patient narratives, surveys or both?. BMC Health Serv Res.

[32] Edwards K, Walker K, Duff J (2015). Instruments to measure the inpatient hospital experience: a literature review. Patient Exp J.

[33] Ranard BL, Werner RM, Antanavicius T, Schwartz HA, Smith RJ, Meisel ZF, Asch DA, Ungar LH, Merchant RM (2016). Yelp reviews of hospital care can supplement and inform traditional surveys of the patient experience of care. Health Aff.

[34] Beattie M, Murphy D, Atherton I, Lauder W (2015). Instruments to measure patient experience of healthcare quality in hospitals: a systematic review. Syst Rev.

[35] Farrington C, Burt J, Boiko O, Campbell J, Roland M. Doctors' engagements with patient experience surveys in primary and secondary care: a qualitative study. Health Expect. Apr 28,2016. 10.1111/hex.12465. 10.1111/hex.12465 PMC5433536 27124310

[36] Williams KE, Sansoni J, Morris D, Thompson C. A Delphi study to develop indicators of cancer patient experience for quality improvement. Support Care Cancer Jul2017. 10.1007/s00520-017-3823-4. 10.1007/s00520-017-3823-4 28711975

[37] Cancer Australia. National Cancer Control Indicators; Cancer control continuum; patient experience. 2017. https://ncci.canceraustralia.gov.au/psychosocial-care/patient-experience. Accessed 6 Dec 2018.

[38] Bureau of Health Information. Patient perspectives – hospital care for Aboriginal people. 2016 Sydney (NSW). http://www.bhi.nsw.gov.au/__data/assets/pdf_file/0010/323929/patient-perspectives-hospital-care-for-aboriginal-people-report-2016.pdf. Accessed 6 Dec 2018.

[39] Green M, Cunningham J, O'Connell D, Garvey G. Improving outcomes for Aboriginal and Torres Strait Islander people with cancer requires a systematic approach to understanding patients' experiences of care. Aust Health Rev. 2017;41(2):231–3. 10.1071/AH15214 27385494

[40] Yerrell PH, Roder D, Cargo M, Reilly R, Banham D, Micklem JM, Morey K, Stewart HB, Stajic J, Norris M, Brown A, Roslyn WA, Cynthia WA, Miller S, Burgoyne A, Fazulla N, Kenmore G, Mitchell A, Keefe D, Eckert M, Farshid G (2016). Cancer data and Aboriginal disparities (CanDAD)—developing an advanced Cancer data system for Aboriginal people in South Australia: a mixed methods research protocol. BMJ Open.

[41] Aboriginal Health and Medical Research Council. Guidelines for Research into Aboriginal Health, Key Principles. 2016. Superseded by: National Health and Medical Research Council, Ethical conduct in research with Aboriginal and Torres Strait Islander Peoples and communities: Guidelines for researchers and stakeholders (2018), Commonwealth of Australia: Canberra. Accessed 6 Dec 2018.

[42] Australian Institute of Aboriginal and Torres Strait Islander Studies. Guidelines for ethical research in Australian Indigenous studies. 2012. https://aiatsis.gov.au/sites/default/files/docs/research-and-guides/ethics/gerais.pdf. Accessed 6 Dec 2018.

[43] Jamieson LM, Paradies YC, Eades S, Chong A, Maple-Brown L, Morris P, Bailie R, Cass A, Roberts-Thomson K, Brown A. Ten principles relevant to health research among Indigenous Australian populations. Med J Aust. 2012;197(1):16–8. 10.5694/mja11.11642 22762218

[44] National Health and Medical Research Council. Values and Ethics: Guidelines for Ethical Conduct in Aboriginal and Torres Strait Islander Health Research. 2003.

[45] Quality Health Ltd. National Cancer Patient Experience Survey. 2014. Retrieved from https://www.qualityhealth.co.uk/resources/surveys/national-cancer-experience-survey/2014-national-cancer-patient-experiencesurvey/2014-national-cancer-patient-experience-survey-materials/689-2013-national-cancer-patient-experiencesurvey-questionnaire-pdf/file. Accessed 6 Dec 2018.

[46] Victorian Comprehensive Cancer Centre (VCCC) 2017. https://www.victorianccc.org.au/news/vccc-cancerpatient-experience-survey-results/. Accessed 6 Dec 2018.

[47] Ipsos Social Research Institute. Development Report: 2014 Adult Admitted Patient Survey. Sydney (NSW); 2015. http://www.bhi.nsw.gov.au/__data/assets/pdf_file/0019/320491/patient-survey-adult-development-report-2014.pdf. Accessed 6 Dec 2018.

[48] Janca A., Lyons Z., Balaratnasingam S., Parfitt D., Davison S., Laugharne J. Here and Now Aboriginal Assessment: background, development and preliminary evaluation of a culturally appropriate screening tool. Australas Psychiatry 2015 Jun;23(3):287–292. 10.1177/1039856215584514. Epub 2015 May 4. 10.1177/1039856215584514 25944764

[49] Campbell JL, Quincy C, Osserman J, Pedersen OK (2013). Coding in-depth Semistructured interviews: problems of unitization and Intercoder reliability and agreement. Sociol Methods Res.

[50] Laycock A, with Walker D, Harrison N, Brands J. Researching Indigenous health: a practical guide for researchers. The Lowitja Institute, Melbourne https://www.lowitja.org.au/sites/default/files/docs/Researchers-Guide_0.pdf. Accessed 6 Dec 2018.

[51] Australian Human Rights Commission. Face the Facts. 2008. https://www.humanrights.gov.au/publications/2008-face-facts-chapter-1#fnB60. Accessed 6 Dec 2018.

[52] Wong ST, Haggerty J. Measuring Patient Experiences in Primary Health Care. 2013. Centre for Health Services and Policy Research, University of British Columbia. https://open.library.ubc.ca/cIRcle/collections/facultyresearchandpublications/52383/items/1.0048528. Accessed 6 Dec 2018.

[53] Knaul F, et al. Alleviating the access abyss in palliative care and pain relief-an imperative of universal health coverage: the Lancet Commission report. Lancet. 2017. 10.1016/s0140-6736(17)32513-8. 10.1016/S0140-6736(17)32513-8 29032993

[54] Cognetta-Rieke C, Guney S (2014). Analytical insights from patient narratives: the next step for better patient experience. J Patient Exp.

[55] Australian Institute of Health and Welfare. National best practice guidelines for collecting Indigenous status in health data sets. Cat.no.IHW29.2010. Canberra, Australia.

